# The Rise of Functional Tic-Like Behaviors: What Do the COVID-19 Pandemic and Social Media Have to Do With It? A Narrative Review

**DOI:** 10.3389/fped.2022.863919

**Published:** 2022-07-11

**Authors:** Jaclyn M. Martindale, Jonathan W. Mink

**Affiliations:** ^1^Department of Neurology, Wake Forest University School of Medicine, Atrium Health Wake Forest Baptist, Winston-Salem, NC, United States; ^2^Department of Neurology, University of Rochester Medical Center, Rochester, NY, United States

**Keywords:** tourette, tic, functional tic, functional movement disorders, mass psychogenic illness, TikTok, COVID-19, tic-like behavior

## Abstract

**Background:**

There has been a rise in explosive onset of tic-like behaviors during the COVID-19 pandemic. Historically, this is an uncommon phenomenology of functional movement disorders across all ages. Both the psychological burden of the pandemic and social media usage have been implicated in the rise of these tic-like behaviors.

**Methods:**

This paper provides a narrative review of the literature on chronic tic disorders, functional tics, and mass functional illness with particular focus on the key distinguishing features, role of social media, and the role of COVID-19.

**Results:**

The COVID-19 pandemic has profoundly affected the mental health of many individuals, including children, adolescents, and their caregivers. Implementation of lockdowns, lifestyle disruptions, school closures, and social distancing have driven a surge in social media and digital technology use. The combination of predisposing factors, the psychological burden of the COVID-19 pandemic, and social media are implicated in the rise and spread of tic-like behaviors; which may represent a modern-day form of mass functional illness. While many of the features overlap with functional tics, there are emerging distinctive features that are important to recognize. A more encompassing term, *Functional Tic-Like Behaviors*, is used to better reflect multiple contributing factors.

**Conclusion:**

Knowledge of these differences is essential to mitigate downstream health effects and poor outcomes.

## Introduction

In December 2019, a novel coronavirus (COVID-19) was discovered in Wuhan, China. By March 11, 2020, the World Health Organization (WHO) declared a global pandemic of severe acute respiratory syndrome due to coronavirus-2 (SARS-CoV-2)^1^. This led to implementation of lockdowns, lifestyle disruptions, school closures, and social distancing. To date, there have been 246 million confirmed cases and 4.9 million deaths from COVID-19 worldwide^2^.

There is mounting evidence of potential neurological and psychological sequela of COVID-19. Peripheral and central neurological complications of COVID-19 infections have reported (1–5) as have rising rates of stress, anxiety, depression, and behavioral problems (6).

During this time, there has been an increase in functional tics (FT) and functional tic-like behaviors (FTLB). Abrupt onset, atypical progression of symptoms, poorly localized premonitory sensation, high-degree of suggestibility, lack of suppressibility, and complete distractibility help distinguish FT from chronic tic disorders (CTD), including Tourette Syndrome (TS). Previously an uncommon phenomenology of functional movement disorders (FMD) (7–11), the rise in FTLB presents an opportunity to understand the overlapping and distinguishing features of this disorder from CTD and FT. Both social media and the psychological burden of the COVID-19 pandemic have been implicated in this increase.

As research in this area is rapidly evolving, the purpose of this article is to provide a narrative summary of our current understanding of FT, FTLB, and mass functional illness, with particular focus on the distinguishing features, social media, and the role of COVID-19. Early recognition of FT and FTLB is essential for improved outcomes (12, 13).

## Methods

**Figure 1:**
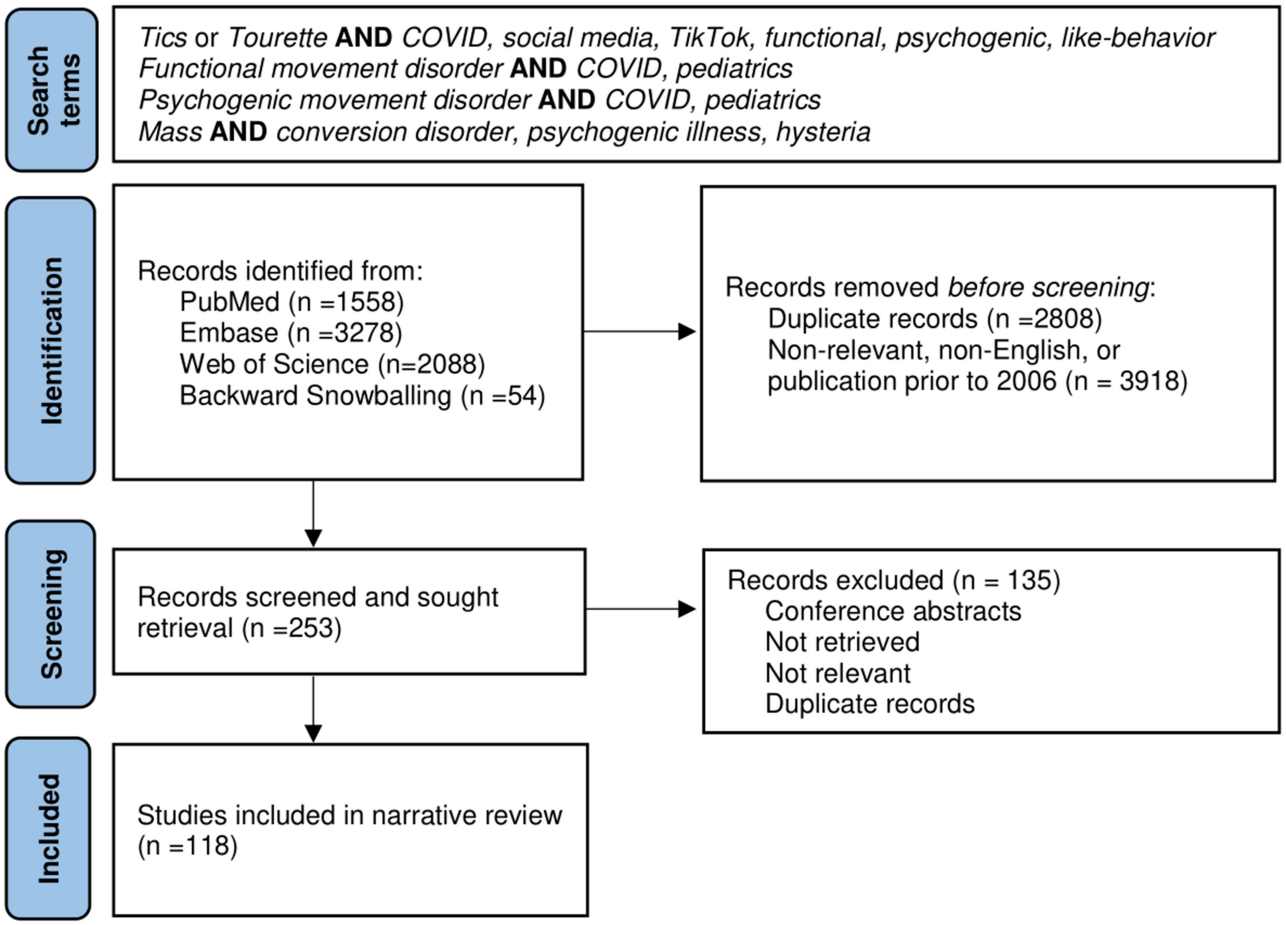
Modified Patient Reporting Items for Systematic Reviews and Meta-Analyses (PRISMA) Figure.

A narrative review was chosen as the synthesis method due to rapidly evolving research in this area. The literature search was conducted using PubMed, Embase, and Web of Science between 2006 through 2021. A modified Patient Reporting Items for Systematic Reviews and Meta-Analyses (PRISMA) figure is included (Figure 1). A combination of search terms produced 6,925 results. Initial search terms included “tic” or “tourette” and “COVID,” “social media,” “TikTok,” “functional,” “psychogenic,” or “like-behavior.” Both “functional” and “psychogenic movement disorders” in context of COVID and pediatrics were also searched. Additionally, the various names for mass functional illness were searched. Inclusion criteria included English literature with publication dates between 2006 and 2021. No specific type of study or age range were excluded from the search. Use of the term functional yielded many non-relevant results related to functional imaging, disability, and anatomy. After removal of duplicates, non-relevant, and non-English literature, as well as backwards snowballing, 118 articles were included for final review. Given the topic of social media, additional publicly available content was included in the review as noted in the footnotes.

### Chronic Tic Disorders

Tics are sudden, rapid, non-rhythmic movements or vocalizations, which occur in 1 in 5 school-aged children (14). Chronic Tic Disorders (CTD), including Tourette Syndrome (TS), are developmental neurobiological disorders characterized by multiple motor and/or vocal tics for at least 1 year. Co-occurring conditions such as attention deficit hyperactivity disorder (ADHD), obsessive-compulsive disorder (OCD), and anxiety occur in 90% of individuals with CTD (15–19).

Tics begin gradually in early childhood, fluctuate, and change over time (16, 18, 20). There is a male predominance of tics, 4:1 male to female ratio, which diminishes in adulthood (18, 20, 21). For the vast majority of individuals with CTD, tics peak in the peri-pubertal period and improve through adolescence (20).

Tics often begin as simple motor tics and progress over time in a rostro-caudal distribution (22). Tics are preceded by a premonitory urge, often localized to the affected body region (23). Premonitory urges are an itch, tension, need or unpleasant sensation that builds with voluntary suppression and is relieved by completing the tic (24, 25). Recognition of premonitory urges tends to occur between 8 and 10 years old, although is reported by the majority of individuals with CTD (24, 26, 27).

Whether the phenotype of TS is different in affected females is poorly understood. There are conflicting reports on the sex-differences of comorbidity prevalence (16, 18, 21, 28–32). Some studies have reported TS-affected females have later onset of tics (16, 33, 34), later peak of tic severity (29, 34), motor tic severity (32, 33), and lower likelihood of tic remission; however, others have shown conflicting results or no significant sex-differences in these factors (18, 28, 32, 33, 35).

Complex tics, including coprophenomena, echophenomena and self-injurious behaviors (SIB) are often misunderstood by the public (36). Risk of coprophenomena such as obscene gestures (copropraxia) or words (coprolalia) increases with age and co-occurring conditions. Coprolalia is three times more common than copropraxia, with a lifetime prevalence of 8–18.5 and 5.7% respectively and often occurs within 5 years of tic onset (18, 37–39).

Echophenomena refers to the repetition of other's actions (echopraxia) or sounds/words (echolalia). In the original publications from Georges Gilles de la Tourette, the persistence of echophenomena beyond normal expected childhood development was essential for the diagnosis of TS (40). Given the heterogeneity of CTD, echophenomena are now considered a distinctive feature rather than a requirement for diagnosis (41), with an estimated lifetime prevalence 43–56% (42, 43).

SIB can occur in 4–53% of individuals with CTD (18, 44, 45). Estimates vary depending on the definition of SIB, which can range from skin picking or scratching, biting, head banging, or self-hitting, to more severe symptoms such as self-cutting, body deformation, or self-mutilation (46). Rarely, individuals affected with TS can have life-threating symptoms (44, 47).

### Functional Movement Disorders

Functional movement disorders (FMD) are a common presentation in neurology clinics (48). Many different terms have described these disorders including conversion disorder, psychogenic, nonorganic, medically unexplained, and hysteria (49, 50). Although *psychogenic* and *functional* are often use interchangeably, *functional* is the preferred terminology. Functional is freer of stigma and more reflective of the current understanding of the pathophysiology on FMD, which suggest a neurobiological basis of these disorders (12, 49, 51, 52). A combination of predisposing, precipitating, and perpetuating factors play a role in development of FMD. Psychosocial stressors, low socioeconomic status, psychiatric comorbidities, female gender, and adverse experiences may increase risk of FMD. It is hypothesized that both epigenetic and genetic factors may contribute to FMD, but current evidence is limited (53). Additionally, brain maladaptation and plasticity may serve as perpetuating factors for FMD (12, 49, 54–56). The Diagnostic and Statistical Manual for Mental Disorders–Fifth Edition (DSM-5) adopted new emphasis on diagnosing based on positive features and removed the necessity of a precipitating stressor as with many patients none are found (57).

### Epidemiology of Functional Tics

The true prevalence of FMD is hard to discern given diagnostic uncertainty, inconsistent terminology, and variability of utilized billing codes (48, 58). Diagnosis relies on inconsistencies and incongruences with known movement disorders. Across all ages, tremor, dystonia and gait disorders are the most common phenomenology of FMD, and FT were rarely reported (13, 59–61).

Estimates of pediatric FT prevalence vary from 0 to 17% (61–64). The rarity of FT may be attributed to the challenge in distinguishing FT from CTD. Many of the positive features used to diagnose FMDs such as distractibility, suggestibility, and fluctuating course are common amongst CTD. Clinical expertise and prior case studies suggest there are some key distinguishing features (10, 12, 65–68). While most individuals with pre-existing FMD reported no change in symptoms with the COVID-19 pandemic, there has been a dramatic increase in new FT (7–11, 69, 70).

### Clinical Phenomenology of Functional Tics

FT have key clinical characteristics that distinguish them from CTD. FT present in adolescents often without a prior history of tics. There is a 3:1 to 9:1 female predominance in FTs. This is in contrast to CTD, which are heavily male dominant (13, 20, 59, 61, 71). Common features include abrupt onset followed by a static or progressive course, high-degree of suggestibility, and complete distractibility. FT lack suppressibility, build up with voluntary suppression, or relief upon completion of the tics (65, 66). Although the presence or absence of a premonitory urge is less definitive, when present in FT it is less often localized to the area of the tic-like behavior (11, 72, 73).

Unlike CTD, FT include complex and large amplitude movements at onset (11). Complex tics such as pali-, echo-, and copro-phenomena are less common in FT and are often more complex, variable, longer in duration, or context-dependent (72, 74, 75). The progression of FT tends to disregard the expected rostro-caudal distribution seen in CTD (66, 76). Frequently other functional neurological or somatic symptoms are present (65, 66).

Some studies note a lack of family history of tics in individuals with FT; however, an important caveat is that there may be a heredity component to FMD (51, 65, 77, 78). There is also potential for false-negative family history, as some may not recognize they had tics previously. Alternatively, a false-positive family history can occur given the prevalence of CTD. Additionally, while a precipitating event can occur, it is important to note stressors and adverse experiences are risk factors rather than requirements for diagnosis (13, 50, 64, 65). Treatment resistance to typical tic medications may also occur (65, 66).

### Mass Functional Illness

Mass Functional Illness (MFI), also known as mass psychogenic illness, mass hysteria, mass conversion disorder, or mass sociogenic illness, is “the rapid spread of illness signs and symptoms affecting members of a cohesive group” (79). MFI has been described for many centuries and occurs in varied cultures, ethnic groups, and religious settings (79, 80).

Historically, there are two categories of MFI: anxiety or motor phenomena. Anxiety MFI is characterized by transient, benign symptoms typically resolving within 24 h when there is a sudden, extreme stress or perceived threat in a cohesive group (81). Symptoms can include dizziness, headache, fatigue or hyperventilation. Motor MFI typically presents with gradual onset of motor symptoms including hyper- or hypo-kinetic movements, gait abnormalities and speech difficulties. Symptoms evolve over weeks to months and gradually remit.

Over the past two decades there has been increased motor presentations. There are many examples throughout recent history of MFI including outbreaks of non-epileptic spells, weakness, twitching, and gait abnormalities often in adolescent females (82–86)^3^.

Perhaps one of the most notable relevant examples was the outbreak of sudden onset of tic-like behaviors in August 2011 through January 2012 at Le Roy High School in Western New York State (68). The 19 affected individuals (18 females, 1 male), who did not belong to the same social group initially, formed a new social group based on their common disorder. Similarly, there were two outbreaks of hiccups and vocal tic-like behaviors of over a dozen students in two nearby Massachusetts high schools in November 2012 and January 2013 (87).

Like FMD, females have been reported to have a higher propensity to MFI (88, 89). A recent meta-analysis of gender differences showed 2.4:1 female predominance of MFI in children and adolescent (90). These outbreaks become the target of substantial media attention as well as thorough investigations into exposures, recent vaccinations, or environmental triggers (90–93).

### Role of Social Media

Presence of movement disorders on social media is not novel. Review of videos on YouTube in 2011 by movement disorder specialists revealed 66% of movement-related videos were FMD (94). Interestingly enough, 18% (5,450/30,095) of movement-related videos reviewed were categorized as tic-related content.

Historically, MFI has been limited to a cohesive group; however, in the modern-day era, social media breaks the geographic barriers that typically confine such symptoms. Bartholomew was one of the first to propose the role of social media in MFI (81, 87). YouTube and Facebook were implicated in the spread of tic-like behaviors in Le Roy, as affected individuals were uploading videos of their symptoms onto these social media sites^4^^,^^5^^,^^6^^,^^7^.

A similar phenomena occurred in Germany in June 2019 when German Neurologists saw a vast rise in FT strikingly similar to a popular YouTube Channel “Gewitter im Kopf [Thunderstorm in the Brain]”, staring a young man Jan Zimmerman (95, 96). The channel gained rapid popularity and has more than 2.2 million subscribers and 312 million views^8^. Zimmerman has a similarly large presence across multiple platforms. Individuals presented with near identical complex movements, vocalizations, and unique words or phrases often seen in Zimmerman's videos. Given the specific role of social media, a more specific term was suggested - mass social media-induced illness (MSMI) (95).

The benefits and risks of social media remain controversial. Some argue that social media and digital technology help maintain social connection despite social distancing and lockdowns (97). Social media can also serve as a platform for individuals to share their experiences, advocate, and educate about medical conditions including tics. However, drawing attention to tics and/or exposure to other's tic-like behaviors may serve as precipitating or perpetuating risk factors for both FT and CTD. While social media provides access to communities that may not be readily available locally, this may also serve as a medium for continued spread of FT. Additionally, overuse of social media is associated with anxiety, depression, and psychological distress all of which may serve as risk factors for FT (98, 99).

### TikTok Tics

Tic-related videos are gaining popularity across social media and the rapid spread of tic-like behaviors is a global phenomenon. On TikTok alone, hashtags of #tourette (4.9 billion views) and #tic (3.1 billion) have grown substantially during the COVID-19 pandemic^9^, hence what some are calling “TikTok Tics”.

TikTok is a popular social media platform where users can create, watch and share short videos. TikTok has experienced a surge in monthly active users between January 2018 and August 2020. Globally TikTok's active monthly users has grown from 54 million users in January 2018 to over 1 billion users as of September 2021^10^^,^^11^. For comparison of active monthly users across other social media platforms Facebook has 2.9 billion, YouTube 2.3 billion, WhatsApp 2 billion, Instagram 1.4 billion, Snapchat 538 million, and Twitter 436 million^12^.

It is important to note that tic-related videos have grown substantially across multiple social media platforms and are not exclusive to TikTok. For example, TikTok influencer Evie Meg, better known as @thistrippyhippie, has 14 million followers for her tic-like behavior but also 791k followers on Instagram and 25 million views on YouTube^13^^,^^14^^,^^15^. Her videos often feature complex movements, coprophenomena, unique triggers and context-dependent tics. This influencer discloses her diagnosis of FND and features other videos of functional dystonia and psychogenic non-epileptic spells (PNES).

Two studies assessed the phenomenology of tic-like behavior on TikTok based on expert review. Both studies found a high degree of coprophenomena, context-dependence, aggression toward others, and self-injurious behavior (7, 100). Tic-like behaviors were highly variable and nonstereotyped. While tic-like behavior often involved the face and neck, there was a higher percentage of movements involving arms or body. Tic severity was overall severe with a high degree of tic attacks reported. There was a female predominance with 64.3% female, 17.6% male, and 14.3% nonbinary based on self-report of gender identity in the user's profile (7). Mean age reported was 18.8 years old although limitations included lack of age disclosure as well as unclear timing of video to onset of symptoms (7).

While these descriptive analyses are important to exploring the relationship of social media and tic-like behaviors, these conclusions were based on observations of social media videos rather than in-depth in-person evaluations. Additionally, negative portrayals of CTD are more popular on social media (101) and may influence the phenomenology reviewed by these two studies.

There has been some question of secondary gain in use of social media. Many of these social media influencers have merchandise for purchase (7). Jan Zimmerman sells merchandise, a book and recently released a Google app with his most popular vocal tics including “tics of the month.” Evie Meg released her new book “My Non-Identical Twin: What I'd like you to know about living with Tourette's” (7, 102, 103). It is important to note that factitious disorders and malingering are distinctly different from FMD and are beyond the scope of this article.

### Functional Tic-Like Behaviors

**Figure 2:**
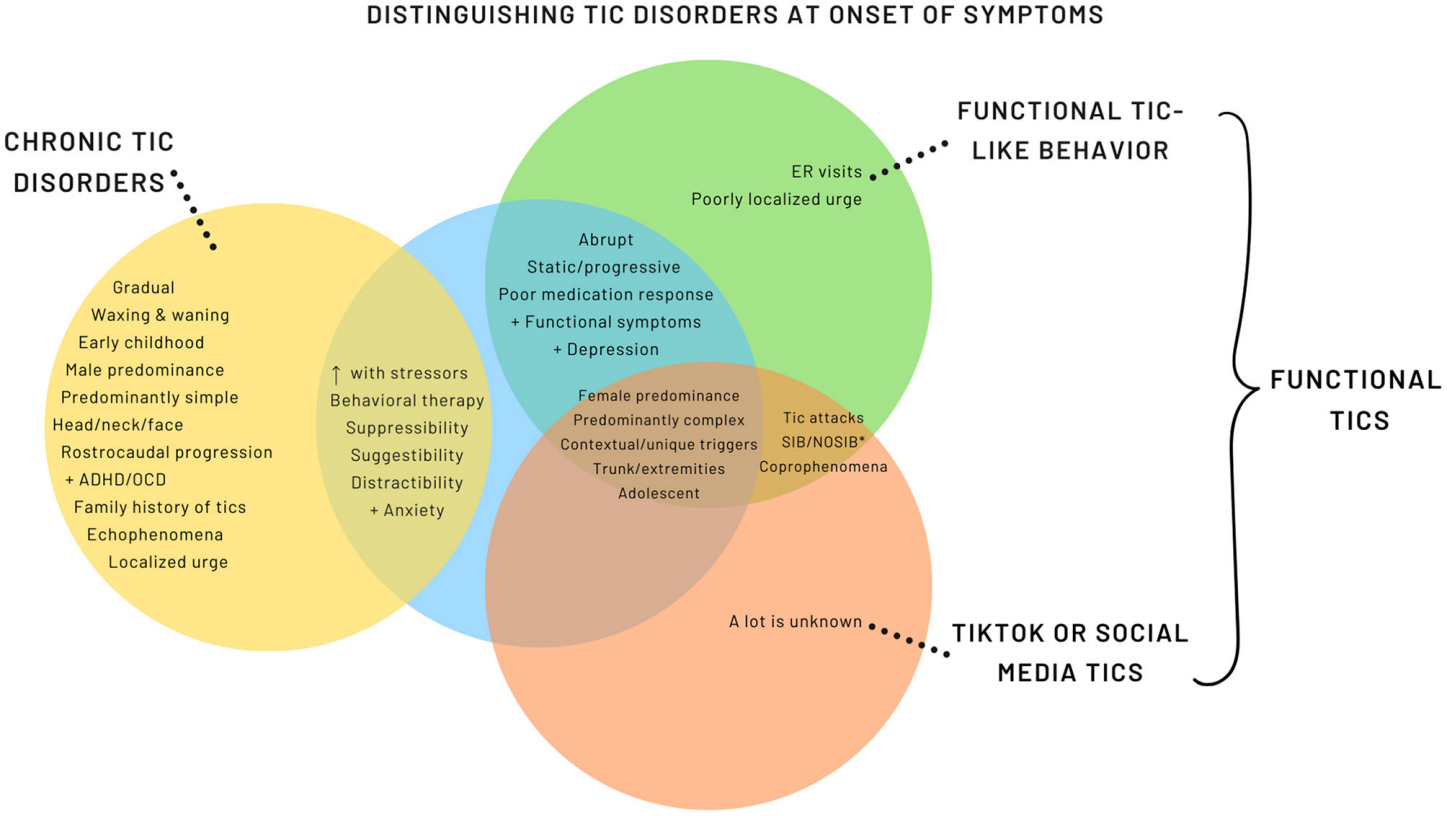
Distinguishing Tic Disorders at Onset of Symptoms. *SIB, Self-injurious behavior; NOSIB, Non-obscene socially inappropriate behavior.

While many of the features overlap with FT, there are emerging distinctive features (Figure 2, Supplementary Table 1). A more encompassing term is used, *Functional Tic-Like Behaviors* (FTLB) to better reflect the combined role of social media and the pandemic.

FLTB have a female predominance (11) with the exception of one report from Germany (96), which had a male predominance. It is possible that this is related to the German social media influencer previously discussed. Median age of FTLB onset ranges from 14.2 to 15.3 years old with initial presentations being abrupt onset, non-fluctuating, and predominately complex tic-like behavior (11, 96, 104). Studies note a higher prevalence of tic-like behavior involving the trunk and extremities relative to the expected rostro-caudal progression seen in CTD (11, 96, 104). Pringsheim et al. reported a higher proportion of anxiety and depression diagnoses in FTLB compared to primary tic disorders (11), whereas others have found no significant difference (96).

FTLB are associated with high prevalence of coprophenomena, odd words or phrases, self-injurious (SIB) and non-obscene socially inappropriate behavior (NOSIB) (7, 71). Additionally, unique or contextual triggers such as particular words, flashing lights, or loud noises can trigger the tics or tic attacks (96, 104). Common SIB include hitting, punching or slapping one's self. Conversely, NOSIB can present as hitting others, throwing or hitting objects (11, 104). Tic attacks and presence of other functional or somatic symptoms were commonly reported (104). There are more limited data and variability in the degree of suppressibility, family history of tics, and presence of premonitory urge in patients with FTLB (11, 96). Careful questioning may endorse exposure to tic-related videos on social media; however, it should be considered one of many risk factors and is not always found (11, 96).

Although the predominance of adolescent females is reported, other sociodemographic features associated with FTLB remain unknown. Further exploration of risk factors and social determinants of health would be useful for prevention and intervention planning.

### Role of the COVID-19 Pandemic

There is mounting evidence on the neurological sequela of COVID-19 infections including new development of movement disorders. Although one may consider post-infectious or infectious phenomena of COVID-19, a recent review of de novo movement disorders related to COVID-19 infections did not report any cases of new tics or tic-like behavior (105).

However, the COVID-19 pandemic has profoundly affected the mental health of many individuals, including children and adolescents. Nearly 168 million children globally missed an entire year of school due to COVID-19 according to the United Nations Education, Scientific and Cultural Organization (UNESCO). In April 2020, 1.5 billion learners were affected by school closures in 195 countries and as of November 2021, 55 million learners were still impacted by school closures with lower socio-economic statuses disproportionally affected (106)^16^^,^^17^.

Prior studies demonstrated both short- and long-term psychological effects of pandemics/epidemics including increased post-traumatic stress symptoms, anxiety, depression, helplessness, and risky behaviors (107–113). The overall rates of depression and anxiety are higher during COVID-19 than prior pandemics (114), with increased risk in females, adolescents, and remote learners (106, 107, 114–121). Periods of intense stress, such as the pandemic, can be associated with increased functional symptoms (122–124).

Parental stress, mental health, and wellbeing are also impacted during the pandemic, which is associated with poorer child wellbeing (125–128). Disruptions from the pandemic altered diets, sleep schedules, and social relationships. Additionally, parents reported interrupted access to medical care and to their support networks. Parents and/or caregivers suffered from isolation, employment changes, food insecurity, housing instability, and financial constraints all while balancing remote-learning and their child's wellbeing (125). Lower socioeconomic status, younger parents, and families of healthcare workers have been reported to be at increased risk of poorer wellbeing (129, 130). Additionally, there are increased reports of childhood adverse experiences during the pandemic, such as witnessed domestic violence, emotional abuse, and physical abuse (131, 132).

There has been an overall increase in new FMD presenting to neurology clinics during the COVID-19 pandemic (10). Despite this increase, individuals with preexisting FMD did not show significant variability or worsening of their symptoms during the pandemic (133). However, up to two-thirds of parents or individuals reported worsening of CTD symptoms (134, 135). Children with neurodevelopmental disorders, such as CTD, report higher behavioral and psychological impacts of the pandemic compared to peers (136). The same is true for children with preexisting mental health diagnoses (125). Acute psychosocial stressors, routine disruption, and increased mental health burden likely play a role in symptom exacerbation or development; however, these relationships need to be further explored (137).

## Discussion

Knowledge of these disorders are vital in mitigating downstream health effects and poor outcomes. A common concern in FMD is fear of misdiagnosis; however, in the modern medical setting the frequency of misdiagnosis is consistently low (138, 139). Recognition of the positive features to support a diagnosis of FMD is essential. While behavioral therapy is the first line treatment for both FMD and CTD, it is critical to establish the diagnosis early and engage familial support (12, 140). Longer duration of symptoms before diagnosis and pre-existing personality disorders lead to poorer outcomes (141, 142). A multidisciplinary approach is essential in effective treatment and psychological support is crucial. The overall mental health burden of the pandemic poses challenges for accessibility to knowledgeable therapists and mental health resources.

Clinicians should also be mindful that FTLBs may co-occur in individuals with CTD or other neurological conditions (143). A sudden or explosive emergence of atypical tic-like behaviors should raise concern of functional overlay (144, 145). Failure to recognize this can lead to unnecessary medication trials, sense of pseudo-refractoriness, potential invasive surgical procedures or delay in diagnosis (61, 146, 147).

The role of social media in these tic-like behaviors has gained significant media attention, which likely contributes to parental fear and uncertainty. Explosive onset of FTLB can be both bothersome and intrusive to the daily function of the individual and their family. This may result in missed days at school, parental missed days of work, missed social events, and/or financial constraints that impacts parental stress and wellbeing. Many patients with explosive onset of FTLB are utilizing emergency room services (9). FMD admissions have higher work-up rates but shorter length of stays. In 2017, the estimated US economic impact of ER and inpatient care of FNDs was more than $1.2 billion annually, comparable to other high-utilization neurological conditions such as refractory epilepsy or demyelinating disorders (58). Recognition of FTLB may reduce unnecessary admissions, diagnostic testing, medication trials, time to treatment, and economic impacts.

The impact this global phenomena has had on the CTD community must also be considered. A look through the comments on these influencers' videos suggests a step backwards in awareness, attitudes, and stigmatization of not only CTD but also FMD community. In CTD, female gender, tic severity and complex tics increase stigmatization risk, which is associated with lower quality of life, depression, and lower self-esteem (36, 148–151). The commonality of these features with FT and FTLB may contribute to ongoing public misconception of individuals with CTD and FMD. Future research should aim to understand the intricacies of stigmatization in these disorders.

Lastly, the rarity of FT previously may have limited our understanding of this disorder. With the rise of FTLB, there is an opportunity to evaluate overlapping and distinguishing features of FT, FTLB, and CTD to establish evidence-based guidelines for evaluation and treatment. Previous studies have suggested some common predisposing factors between CTD and FT such as family history, adverse experiences, and psychosocial stressors (11, 72). Lastly, the etiology of FLTB is likely multifactorial. Future research is necessary to better define the relationship between social media, the pandemic, and these entities as well as further understand shared predisposing, precipitating, and perpetuating factors.

## Author Contributions

JMM: conceptualized, drafted the initial manuscript, reviewed, and revised the manuscript. JWM: conceptualized, critically reviewed the manuscript for important intellectual content, and revised the manuscript. All authors approved the final manuscript as submitted and agree to be accountable for all aspects of the work.

## Conflict of Interest

The authors declare that the research was conducted in the absence of any commercial or financial relationships that could be construed as a potential conflict of interest.

## Publisher's Note

All claims expressed in this article are solely those of the authors and do not necessarily represent those of their affiliated organizations, or those of the publisher, the editors and the reviewers. Any product that may be evaluated in this article, or claim that may be made by its manufacturer, is not guaranteed or endorsed by the publisher.

## Footnotes

^1^World Health Organization. (2020). Director-General media briefing on COVID-19. https://www.who.int/director-general/speeches/detail/who-director-general-s-opening-remarks-at-the-media-briefing-on-covid-19-−11-march-2020 (Accessed 11/4/2021).

^2^World Health Organization. (2020). Coronavirus (COVID-19) Dashboard. https://covid19.who.int/ (Accessed 11/2/2021).

^3^The New York Times. (2007). Mysterious illness strikes teenage girls in Mexico. https://www.nytimes.com/2007/04/16/world/americas/16iht-mexico.3.5306132.html (Accessed 10/26/2021).

^4^The New York Times Magazine. (2012). What Happened to the Girls in Le Roy. https://www.nytimes.com/2012/03/11/magazine/teenage-girls-twitching-le-roy.html (Accessed 10/26/2021).

^5^The Daily Mail. (2012). Facebook to blame for the panic surrounding mysterious Tourette's-like illness spreading in rural New York town. https://www.dailymail.co.uk/news/article-2096813/Could-infection-mysterious-Tourettes-like-syndrome-affecting-teenagers.html (Accessed 10/26/2021).

^6^TODAY. (2012). Facebook, YouTube could be spreading 'mystery illness,' doctor says. https://www.today.com/health/facebook-youtube-could-be-spreading-mystery-illness-doctor-says-1C9381793 (Accessed 10/26/2021).

^7^Huffpost. (2014). When Social Media Makes Something Go Viral In Real Life. https://www.huffpost.com/entry/dont-look-now-social-medi_b_5534200 (Accessed 10/26/2021).

^8^YouTube. (2005). Gewitterimkopf. https://www.youtube.com/c/gewitterimkopf/about (Accessed 10/28/2021).

^9^TikTok. (2016). Tag: Tourette's. https://www.tiktok.com/tag/tourettes?lang=en (Accessed 10/28/2021).

^10^CNBC. (2021). TikTok says 1 billion people use the app each month. https://www.cnbc.com/2021/09/27/tiktok-reaches-1-billion-monthly-users.html (Accessed 10/27/2021).

^11^TikTok. (2021) TikTok Newsroom: Thanks a billion!. https://newsroom.tiktok.com/en-us/1-billion-people-on-tiktok (Accessed 10/27/2021).

^12^Datareportal. (2021) Global Social Media Stats. https://datareportal.com/social-media-users (Accessed 10/27/2021).

^13^Social Blade. (2018). User Summary: This Trippy Hippie - YouTube. https://socialblade.com/youtube/channel/UCJHvN0zYgO2ZLjePERRLjKQ (Accessed 10/28/2021).

^14^Social Blade. (2018). User Summary thistrippyhippie – TikTok. https://socialblade.com/tiktok/user/thistrippyhippie (Accessed 10/28/2021).

^15^Social Blade. (2018) User Summary eviemeg – Instagram. https://socialblade.com/instagram/user/eviemeg (Accessed 10/28/2021).

^16^UNESCO. (2021). Education: From disruption to recovery. https://en.unesco.org/covid19/educationresponse#schoolclosures (Accessed 11/01/2021).

^17^UNESCO. (2020). 1.3 billion learners are still affected by school or university closures, as educational institutions start reopening around the world, says UNESCO. https://en.unesco.org/news/13-billion-learners-are-still-affected-school-university-closures-educational-institutions (Accessed 11/1/2021).

## Abbreviations

COVID-19: coronavirus disease 2019
SARS-CoV-2: severe acute respiratory syndrome due to coronavirus-2
FMD: functional movement disorder
FT: functional tics
FND: functional neurological disorder
CTD: chronic tic disorders
TS: Tourette Syndrome
ADHD: attention deficit-hyperactivity disorder
OCD: obsessive-compulsive disorder
MFI: mass functional illness
PNES: psychogenic non-epileptic spells
MSMI: mass social media-induced illness
UNESCO: United Nations Education, Scientific and Cultural Organization.
