# Supplementary material for: The Rise of Functional Tic-Like Behaviors: What Do the COVID-19 Pandemic and Social Media Have to Do With It? A Narrative Review
Source: Front Pediatr. 2022 Jul 11;10:863919. doi: 10.3389/fped.2022.863919 (PMC9309505; doi:10.3389/fped.2022.863919)
Supplement: Supplementary file 1 [file Table_1.docx]

Supplementary Material

# Supplementary Figures and Tables

| Supplementary Table 1: Summary of Clinical Features of Tic Disorders | | | | |
| --- | --- | --- | --- | --- |
|  | **Chronic Tic Disorders** | **Functional Tics** | **TikTok or Social Media Tics** | **Functional Tic-Like Behaviors** |
| Symptom onset | Gradual | Abrupt | Unknown | Abrupt |
| Course | Waxing and waning | Static or progressive | Unknown | Static or progressive |
| Age of onset | Early Childhood | Adolescence | Unknown, young adult prevalence on videos | Adolescence |
| Gender predominance | Male | Female | Female | Female |
| Triggers | Stress, excitement, fatigue, attention | Stress, anxiety | **Contextual or unique triggers** | Stress, anxiety, **contextual or unique triggers** |
| Family history of tics | Common | Less common | Unknown | Less common |
| Motor tic complexity at onset | Simple | Complex | Unknown | Complex |
| Vocal tic complexity at onset | Simple | Complex | Unknown | Complex |
| Tic phenomenology at onset | Simple | Mostly complex | Unknown | Mostly complex |
| Tic location | Head, face, and neck | **Trunk and extremities** | **Arms and body** | **Trunk and extremities** |
| Coprophenomena | Uncommon | Uncommon | **Common** | **Common** |
| Echophenomena | Common | Uncommon | Uncommon | Uncommon |
| Premonitory sensation | Common | Uncommon | Unknown | **Mixed, poorly localized** |
| Distractibility | Partial | Completely | Unknown | Completely |
| Suppressibility | Partial, temporary | None/Minimal | Unknown | **Variable** |
| Suggestibility | Partial | Very | Unknown | Very |
| Self-injurious or non-obscene socially inappropriate behavior | Later onset | Uncommon | **Common** | **At onset** |
| Common  Comorbidities | ADHD, OCD, and anxiety | Anxiety, depression, and other functional or somatic symptoms | Unknown | Anxiety, depression, and other functional or somatic symptoms |
| Tic attacks | Rare | Less common | **Common** | **Common** |
| ER visits | Rare | Less common | Unknown | **Common** |
| Response to tic medication | Variable | Poor | Unknown | Poor |
